# Science in Straits

**Published:** 1891-01-31

**Authors:** 


### THE DOCTOR'S ARMCHAIR.

### SCIENCE IN STRAITS.

ALTHOUGH a man may himself be a worker in the fields of science, it is both profitable and mentally entertaining for him to sometimes put down his spades and his wheelbarrows and to seek some friendly hillock from whence he may survey the scientific diggings and delvings of other people. There is no reason why men of science should not furnish instruction to the casual or deliberate spectator, and even a little sober amusement too. Scientists do not like to be laughed at; but that is unfriendly of them, and shows a deficiency of humour. A thoroughly wholesome-hearted man enjoys the fun which may be aroused by something which is accidentally ridiculous in himself as much as the spectator enjoys it; and a really sensible man knows that he can no more be always preter-naturally wise and sober than he can always manage to keep his hair nicely brushed or to have a judicious and dignified balance at his banker's. The wisest, the soberest, the most dignified of men must sometimes inevitably look a little ridiculous, even to the wife of his bosom. When he finds himself in such admittedly trying circumstances, let him yield to the situation, and enjoy the laugh at his own expense as much as he would do if some other grave personage were suddenly to find himself decked in a clown's apparel with well-chalked cheeks and the tip of his nose beautified by an adornment of vermilion. It is a grave defect in a man when he cannot bear to be laughed at; it may almost be said of him as Shakespeare said of the man who was destitute of music. Putting the word "laughter" where "music" ought to be, the familiar quotation will fit him like a glove :—

> The man that hath no laughter in himself,
> * * * * *
> Is fit for treasons, strategems, and spoils:
> The motions of his spirit are dull as night
> And his affections dark as Erebus;
> Let no such man be trusted.

Certain men of science, it seems to us, have been making themselves a little ridiculous lately. We hardly dare hint, as yet, that Koch set the fashion. But the two or three American doctors who so promptly filed claims of priority of discovery have repeated with such unerring accuracy a familiar proceeding in the history of medical progress that it is impossible to resist the temptation to laugh at them. Quite as ridiculous were the two Frenchmen MM. Picq and Bertin in their very inopportune choice of a moment for announcing the superiority of transfused goat's blood as a method of treating consumption. Goat's blood may, for anything we know to the contrary, prove to be the coming remedy for tubercular phthisis; but, at least, it cannot be said to have "arrived" as yet, to use the familiar slang of the day. MM. Picq and Bertin seem to have been fired with an ardent patriotism, which, however admirable it may be when the village drum is being thumped, and all the children and dogs of the parish are rushing after the last new recruit as he proceeds to the railway station to join the awkward squad, is supremely absurd as a counter-demonstration to a scientific discovery made in another country. "Provincialism" should be shunned by men of science with as much horror as a Latin teacher expresses for a false quantity, or as an unquestionable exquisite of the period, according to Lord Esher, flies from an ill-made pair of trousers.

Scientists, in truth, appear to be somewhat in straits at this particular juncture. Original research is the "dark horse" which everybody backs to win; and that youthful or middle-aged scientist who has discovered nothing of an original kind, feels that life is not worth living; he may as well throw up the sponge. Undoubtedly this is an unhappy state of things. When Alexander found that there were no more worlds to conquer he very properly took out his pocket-handkerchief and had a good "blubber:" or did he use his coat-sleeve, being too tightly encased in buckram to get at his pocket-handkerchief? At any rate, we have it on the high authority of unquestioned schoolboy tradition, that he wept: and if anybody suggests that a birch rod would have been a proper remedy to employ for the cure of his troubles, he must be an exceedingly unsympathetic and ill-conditioned person. It has happened to some of our youthful physiologists as it happened to Alexander; and at about the same period of their lives. They and their predecessors have made such brilliant advances that they are coming to the very edge of the physiological planet; and a few steps further, if they do not proceed with caution, may plunge them into the region of the immeasurable—possibly even of the ridiculous.

The passion for knowledge, and for new knowledge, cannot be too ardently admired. We would all give our ears to know everything about the origin and nature of consumption, cancer, diphtheria, and other such diseases; and to have always ready for use some prompt method of treatment which should check them at the outset, drive them out of the patient's system, and enable us to present the endangered person to his friends at the end of a few days or weeks a free, healthy, and happy man. "Our ears," did we say? Some medical men would give their lives for the possession of such knowledge and power. And they would count the gain to medicine cheap at the cost. But, then, although such ardour for new knowledge and power cannot be commended too much, yet every traveller should be able to see when a stone wall a hundred feet high blocks his pathway; and if he does not see it, even though his eyes be open, we call him by an unpleasant name. He ought to know that progress is barred that way, at any rate for the present, and that it should be sought in some other direction.

Now there are two methods of advancing the science and art of medicine—the one is laboratory research; the other is clinical watchfulness, with all that the phrase implies. By the younger men, laboratory research is the method which is almost solely pursued; and the reason is that the applause of the scientific world, of the general public, and even of medical men themselves, is often given for the smallest and most useless discovery in the laboratory, when it is withheld from the largest and most beneficent advance at the bedside. And yet, when mortal man is *in extremis*, it is never the mere laboratory scientists who are called to his aid, but those who have acquired skill and resource by experience in the sick-room.

Is it then our aim to belittle the laboratory? The question is absurd! But we claim that the sick-room is a laboratory; the hospital ward is a laboratory; and even the out-patient department; the dead-house, too, is one of the most important of them all. If Alexander, instead of crying when he found no more worlds to conquer, had marched back to Greece, and tried what he could do for the development, the prosperity, and the happiness of his own country, he might have proved himself a truly great man, and have left a noble example for the imitation of politicians of all time. Instead of that, he played the part of a brilliant flash of lightning, which illuminates for a brief moment and burns up what may be in its way, but which gives neither light nor heat to the world of a permanently beneficial kind. Our advice to the medical scientists of the laboratory is that they check and correct their original researches by original observations at the bedside; and that they inspire and guide their bedside observations by the light of their original researches. The field of fresh discovery is becoming narrower every day, and men are driven to the most desperate straits to find out something new. On the other hand, the field of clinical observation neither knows, nor can ever know, a limit; for every new patient differs in some respect from every preceding patient, and has something new to reveal. The first and highest duty of any man who claims to live under the honourable ægis of the medical sciences is to know diseases as they are; to know the deepest necessities of man; and to be able to relieve them. Whoever falls short of these attainments is still a recruit or a non-commissioned officer, and in no sense a leader and a captain in the battle against disease and death.

